# Neuromodulation on the ground and in the clouds: a mini review of transcranial direct current stimulation for altering performance in interactive driving and flight simulators

**DOI:** 10.3389/fpsyg.2024.1479887

**Published:** 2024-11-28

**Authors:** Kayla S. Sansevere, Nate Ward

**Affiliations:** Tufts Applied Cognition Laboratory, Department of Psychology, Tufts University, Medford, MA, United States

**Keywords:** transcranial direct current stimulation, tDCS, driving simulator, flight simulator, transportation

## Abstract

Transcranial direct current stimulation (tDCS) has emerged as a promising tool for cognitive enhancement, especially within simulated virtual environments that provide realistic yet controlled methods for studying human behavior. This mini review synthesizes current research on the application of tDCS to improve performance in interactive driving and flight simulators. The existing literature indicates that tDCS can enhance acute performance for specific tasks, such as maintaining a safe distance from another car or executing a successful plane landing. However, the effects of tDCS may be context-dependent, indicating a need for a broader range of simulated scenarios. Various factors, including participant expertise, task difficulty, and the targeted brain region, can also influence tDCS outcomes. To further strengthen the rigor of this research area, it is essential to address and minimize different forms of research bias to achieve true generalizability. This comprehensive analysis aims to bridge the gap between theoretical understanding and practical application of neurotechnology to study the relationship between the brain and behavior, ultimately providing insights into the effectiveness of tDCS in transportation settings.

## 1 Introduction

There is robust interest in using non-invasive brain stimulation (NIBS) to characterize and modulate human cognition and behavior (Antal et al., 2022; Berryhill, 2014; Bikson et al., 2018; Dubljević et al., 2014; Wexler, 2017, 2022). NIBS methods have potential applications in demanding scenarios that require cognitive, perceptual, and motor skills, such as driving a car or piloting an aircraft. Both drivers and pilots often encounter challenging situations, like making quick decisions at busy intersections or landing in adverse weather conditions. Performance can also suffer in monotonous situations, such as during partially automated driving (McWilliams and Ward, 2021).

Transcranial direct current stimulation (tDCS) is a prominent NIBS technique that delivers weak electrical currents via scalp electrodes to initiate subthreshold membrane polarization and alter neuronal activity (Nitsche et al., 2008; Nitsche and Paulus, 2000, 2001). The electrical current flows into the brain through the anode, which likely increases cortical excitability through depolarization, and exits through the cathode, reducing excitability via hyperpolarization (Liu et al., 2018). If a specific brain region is involved in a task, modulating neuronal excitability could enhance or inhibit performance on that task (Knotkova et al., 2019). Typical current intensities range from 1 to 2.5 mA, although currents as high as 4 mA may be used (Chhatbar et al., 2017; Khadka et al., 2020; Reckow et al., 2018). Stimulation should be applied for no more than 1 h during a task (online) or before it (offline) (Woods et al., 2016). While online stimulation may be ideal for immediate performance enhancement, the effects of offline tDCS can last after the stimulation ends and may be better suited for investigating longer-term neural changes (Bikson and Rahman, 2013; Martin et al., 2014; Miniussi et al., 2013; Ohn et al., 2008; Stagg and Nitsche, 2011).

Electrodes are positioned according to standardized electroencephalography (EEG) system coordinates. In conventional tDCS, two large sponge electrodes deliver a broad current across various brain regions (Kuo et al., 2013). High-definition tDCS (HD-tDCS) is a significant advancement utilizing smaller electrodes arranged closely together to achieve more focal current flow than conventional tDCS (Alam et al., 2016; Datta et al., 2009; Villamar et al., 2013). Commonly targeted brain regions include the dorsolateral prefrontal cortex (DLPFC) and the primary motor cortex (Dedoncker et al., 2016; Jacobson et al., 2012). The DLPFC is linked to working memory, cognitive control, and decision-making (Barbey et al., 2013; Krawczyk, 2002; MacDonald et al., 2000), while the primary motor cortex is associated with skill acquisition and procedural learning (Karni et al., 1998). Evidence from functional near-infrared spectroscopy (fNIRS) and event-based magnetoencephalography (MEG) indicates an interaction between the prefrontal cortex and primary motor cortex during critical driving maneuvers, such as accelerating and braking, particularly under varying demands (Foy and Chapman, 2018; Geissler et al., 2021; Walshe et al., 2022).

Importantly, tDCS is generally well-tolerated in both healthy individuals and clinical populations (Antal et al., 2017; Aparício et al., 2016; Bikson et al., 2016; Palm et al., 2018). Compared to other NIBS methods like transcranial magnetic stimulation (TMS), tDCS is portable and adaptable for various settings, from remotely supervised clinical trials (Pilloni et al., 2022) to physically demanding activities like sprint cycling (Garner et al., 2021; Huang et al., 2019) and military operations (Brunyé et al., 2020; Nelson and Tepe, 2015). The cognitive and perceptual enhancement effects of tDCS on operator performance and workload have been examined using computer-based tasks like the Multi-Attribute Task Battery (MATB) (Nelson et al., 2016, 2019; Rao et al., 2024), which was developed by the National Aeronautics and Space Administration (NASA) to mirror the complex responsibilities that pilots manage in flight (Santiago-Espada et al., 2011). Other gamified tasks, like NeuroRacer (Hsu et al., 2015) and Space Fortress (Scheldrup et al., 2014), have also been used for testing.

Investigating tDCS in more immersive environments could further clarify its practical applications. Interactive driving (Fisher et al., 2011) and flight (Allerton, 2009; Hays et al., 1992) simulators offer safe, controlled settings that mimic real-life demands (Roberts A. P. J. et al., 2020). Interactive simulators are effective in predicting on-road driving skills (Walshe et al., 2022) and supporting pilot training (Ross and Gilbey, 2023). With ongoing research supporting its effectiveness, tDCS holds promise for widespread use in cognitive and motor task enhancement. Given the consistent interest in tDCS across clinical, empirical, and commercial contexts, its potential applications for performance enhancement in transportation settings are highly relevant and merit investigation.

## 2 Current mini review

This review summarizes and evaluates research on the use of tDCS to modulate driver and pilot performance. We conducted searches for refereed articles on Google Scholar and PubMed using the keywords: “driving” OR “flight” AND “transcranial direct current stimulation (tDCS).” This search yielded nine potentially relevant publications. Our scope included studies that recruited healthy participants from nonclinical samples and used interactive driving or flight simulators. Three studies were excluded from review because they did not meet these criteria (Brunnauer et al., 2018; Burkhardt et al., 2023; Pope et al., 2018). Ultimately, six publications met the criteria for inclusion and were reviewed (see Tables 1, 2).

**Table 1:** Driving simulators and tDCS summary of studies.

Study	Design	Masking	Sample	Sessions	Current	Duration	Montage (surface area)	Sham	Key results
Beeli et al. (2008)	Mixed groups	Not mentioned	*N* = 21 *n*_F3_ = 10 *n*_F4_ = 11	3	1 mA	15 min offline	Anode: F3 or F4 cathode: ipsilateral mastoid anode: ipsilateral mastoid cathode: F3 or F4 (35 cm^2^)	None	Fewer speeding violations and more headway distance from pre-stim to post-stim if anodal than cathodal
Sakai et al. (2014)	Within groups	Single-masked	*N* = 13	3	1.5 mA	20 min online	Anode: F3 and F4 cathode: F4 and F3 (35 cm^2^)	30 s ramp up/30 s ramp down	Fewer lane deviations and more accurate headway distance when anodal than cathodal and sham
Facchin et al. (2023)	Within groups	Not mentioned	*N* = 27	3	2 mA	20 min online	Anode: FC4 cathode: Fp1 (35 cm^2^) anode: FC4 cathodes: Cp4/FT8/AF4/FCZ (6 cm^2^)	10 s ramp up/10 s ramp down	Quicker foot and hand RTs when active than sham; stronger effects when HD than conventional

RTs, reaction times.

**Table 2:** Flight simulators and tDCS summary of studies.

Study	Design	Masking	Sample	Sessions	Current	Duration	Montage (surface area)	Sham	Key results
Choe et al. (2016)	Between groups	Double-masked	*N* = 32 *n*_DLPFC_ = 14 (*n*_active DLPFC_ = 7) *n*_M1_ = 18 (*n*_active M1_ = 10)	4	2 mA	1 h online	DLPFC anodes: F6/FC6 cathodes: Fp2/AF8/AF4 M1 anodes: Cp1/Cp3 cathodes: Fp1/F8/F9 (15.7 cm^2^)	60 s ramp up/60 s ramp down	Smoother landings during sessions 3 and 4 if active than sham over DLPFC only
Mark et al. (2023)	Between groups	Single-masked	*N* = 24 *n*_active_ *=* 12 (*n*_novice active_ = 6) *n*_sham_ = 12 (*n*_novice sham_ = 6)	1	1.5 mA	30 min online	Anode: AF8 cathodes: Fpz/T8 (8 cm^2^)	30 s ramp up/30 s ramp down	Smoother landings if active than sham; stronger effects for novices than experts
Feltman and Kelley (2024)	Mixed groups	Single-masked	*N* = 22 *n*_online_ *=* 12 *n*_offline_ = 10	4	2 mA	Online:2× 10 min Offline: 20 min	Anode: P4 cathode: Fp1 (25 cm^2^)	Online 2× 30 s ramp up/30 s ramp down offline 60 s ramp up/60 s ramp down	More likely to follow glide path when active than sham for online only

DLPFC, dorsolateral prefrontal cortex; M1, motor cortex.

### 2.1 tDCS and driving simulators

In the earliest study, Beeli et al. (2008) examined the effects of tDCS over the DLPFC on driving metrics such as speed, headway distance, and lane positioning. Currently, outcomes measured in driving simulators lack gold standard metrics to define meaningful performance changes that translate to real-world driving. Recently, however, research has proposed two composite factors of driving behavior—vehicle control variability and speed—that include metrics like lane positioning, which also have strong face validity as indicators of safe driving (McManus et al., 2024).

Across three sessions, Beeli et al. (2008) tasked 21 participants (20–30 years, all men) to complete a 3-kilometer drive through a city scene with simulated traffic, lights and signs, and pedestrians. The first session was a baseline drive without tDCS. During the other two sessions, the anode and cathode were positioned unilaterally over the DLPFC in a counterbalanced order. Half of the participants randomly received stimulation over the left DLPFC at scalp coordinate F3, while the other half received stimulation over the right DLPFC at scalp coordinate F4. Stimulation was delivered at 1 mA for 15 min offline. Compared to the baseline, participants exhibited fewer speeding violations and maintained greater headway distance when receiving anodal tDCS over the DLPFC compared to cathodal. There were no observable effects of the hemisphere.

Several methodological considerations in this early study must be addressed. Without a sham condition or adequate blinding, it is difficult to disentangle stimulation effects from experimenter influence and participant bias (Boutron et al., 2007). The most common sham procedure ramps up and down the current at the beginning and end of the protocol to mimic initial cutaneous sensations without lasting effects (Woods et al., 2016). Additionally, a between-groups design introduces random variability (Borghini et al., 2014; Lakens, 2013) and fails to account for individual differences in tDCS effects, which can be influenced by anatomical factors (e.g., skull thickness) and behavioral baselines (Bikson et al., 2012; Datta et al., 2012; Horvath et al., 2014; Kim et al., 2014; Li et al., 2015; Opitz et al., 2015; Splittgerber et al., 2020).

Sakai et al. (2014) conducted a sham-controlled, within-groups, single-masked study to address these limitations. Thirteen participants (~35 years, 11 men) were instructed to maintain a specific headway distance from a lead vehicle over a 22-kilometer route. Participants completed this driving task over three testing sessions. In a counterbalanced order, participants received anodal tDCS over the right DLPFC at F4, cathodal tDCS at F4, and sham. Stimulation was set at 1.5 mA for up to 20 min online. There was less variability in headway distance and lane positioning when anodal tDCS was delivered over the DLPFC compared to cathodal and sham. This finding is consistent with research in other domains showing anodal-excitatory effects, but not cathodal-inhibitory effects, for cognitive tasks involving the DLPFC (Jacobson et al., 2012). One explanation could be that the anode likely enhances neuronal firing in active areas, while the cathode may not sufficiently inhibit firing in highly active states.

The neuromodulation field has significantly advanced in the decade since Sakai et al. (2014) published their work. Facchin et al. (2023) explored the effects of different tDCS electrode montages on driving behavior in the latest driving study. Twenty-seven participants (21–30 years, 14 women) completed three 25-min driving sessions while receiving sham tDCS, conventional tDCS, or 4 × 1 HD-tDCS, where four electrodes surround a center electrode of the opposite polarity (Datta et al., 2009; Kuo et al., 2013). Anodal tDCS was applied over the right frontal eye field (FEF) at 1.5 mA over FC4, an area implicated in visuomotor control (Cameron et al., 2015; Grosbras et al., 2005; Nobre et al., 2000). Given that electrode size and material affect spatial resolution, coupled with the structural-functional connectivity of the human brain (Park and Friston, 2013; Sporns, 2013), the DLPFC may have been incidentally targeted during stimulation.

As many can attest, drivers rarely focus on just car following. To this point, Facchin et al. (2023) manipulated driving task difficulty using two variations of stimulus–response detection tasks commonly used in human factors research (Innes et al., 2021). During the drive, the lead car frequently flashed its brake lights, and road signs appeared at random intervals. Participants were asked to brake in response to the lead vehicle and respond to the road signs. Outcomes measured included lane-keeping position, braking reaction time and accuracy, and road sign reaction time and accuracy. Lane maintenance was unaffected by stimulation. Facchin et al. (2023) found that participants responded more quickly, though not more accurately, to the brake lights and road signs when receiving anodal tDCS over the FEF than sham. More prominent effects for these reaction times emerged when stimulation was delivered with HD-tDCS rather than conventional, suggesting heightened response speed to relevant stimuli. Together, these three driving studies indicate that anodal tDCS over the DLPFC may influence distance perception or judgment, observable as changes in distance or faster response times.

### 2.2 tDCS and flight simulators

Choe et al. (2016) examined the impact of tDCS on skill acquisition and performance across various simulated flight tasks, using scenarios with computer-based simulations that align with Federal Aviation Administration (FAA) Industry Training Standards (FITS) to enhance real-world training relevance (Williams, 2012). Though the performance was tested on flight tasks of varying difficulty, results were only published for the easiest task. Across four sessions, 32 participants (ages 21–64, 31 men) attempted to replicate a landing demonstrated in an instructional video under daylight conditions with complete visibility. Measured outcomes included landing gravitational force (*g*-force), deviations from flight path, vertical speed, and vertical speed variance. While *g*-force assessment captures landing skill at the most challenging and critical phase of flight, the entire approach is considered with path and vertical speed deviations. Learning rates were measured across sessions, within sessions, and between trials or scenarios (5 trials per session).

Choe et al. (2016) treated stimulation application and location as between-group factors. Half the participants received anodal tDCS (2 mA, 1 h online), while the other half received sham. Stimulation was delivered over the right DLPFC (anodes F6/FC6) or the left motor cortex (anodes CP1/CP3). No significant effects emerged for the motor cortex group. In the DLPFC group, there was less variability in landing *g*-force observed during the third and fourth sessions, suggesting that tDCS may be more beneficial for trained tasks over time. Choe et al. (2016) also collected EEG and functional near-infrared spectroscopy (fNIRS) data that suggests that participants who received active tDCS exhibited altered neuronal activity in the DLPFC and motor cortex compared to those who received sham. Interestingly, behavioral outcomes are commonly observed when delivering anodal excitation over the motor cortex, but not cognitive regions like the DLPFC (Jacobson et al., 2012; Tremblay et al., 2014). The broad influence of the DLPFC on cognitive functions, especially when considering varying stimulation parameters, makes predicting specific behavioral outcomes difficult.

This initial flight study was conducted under relatively simple conditions to facilitate task performance. However, more realistic scenarios may include bad weather, a narrow runway, or auditory distractions. Accordingly, Mark et al. (2023) adjusted the workload during landing. Twenty-four glider pilots (ages 18–22, mostly men) were recruited and categorized as novices or experts based on experience. Participants completed three runs in a single session, with a pre- and post-training run flanking a tDCS run. In the training run, participants received either sham or anodal tDCS over the right DLPFC at AF8 (1.5 mA, 30 min online). Feedback about performance was presented after each trial (72 trials in total). Measures included landing *g*-force, landing descent speed, and flair.

Mark et al. (2023) observed significant stimulation effects only for landing *g*-force. Specifically, participants who received active tDCS compared to those who received sham landed more smoothly when comparing pre-training to training and post-training. This skill-learning effect was more pronounced in novices than experts, similar to findings in electronic sports (Toth et al., 2021), suggesting novices may benefit more from tDCS. The study took place in a functional magnetic resonance imaging (fMRI) machine, revealing that active tDCS than sham increases DLPFC activity and enhances connectivity between the DLPFC and cerebellum, a region involved in error-feedback learning (Doya, 1999).

Targeting other brain regions with tDCS, such as the posterior parietal cortex (PPC), which guides the visuospatial orienting of selective attention (Behrmann et al., 2004; Culham and Valyear, 2006; Kravitz et al., 2011; Lo et al., 2019; Rojas et al., 2018), could clarify the brain-behavior relationship in flight skill acquisition. In the most recent study, Feltman and Kelley (2024) recruited 22 pilots (~37 years, all men) to complete a 90-min round-trip flight while receiving anodal tDCS over the PPC at P4 (2 mA, 20 min total) and sham. Stimulation timing was treated as a between-groups condition between groups. Participants in the offline stimulation group received anodal tDCS (2 mA, 20 min) and sham before flight. Those in the online group received sham and anodal tDCS (2 mA) delivered for 10 min at 30 and 60 min into the flight.

Toward the end of each flight leg, an emergency required participants to disengage autopilot and land safely. Altitude, airspeed, and heading were measured throughout the flight, while glideslope (vertical) and localizer (lateral) deviations were recorded during the approach. Significant effects emerged only for glideslope deviations in the online group, with online anodal tDCS associated with better alignment to the glide path than sham. These findings align with the role of the PPC in visuospatial attention. Together, these flight studies suggest that tDCS over the DLPFC and PPC may enhance landing smoothness, each investigating different aspects of stimulation and simulation parameters.

## 3 Discussion

Operating a vehicle requires substantial cognitive, perceptual, and motor resources. Non-invasive neuromodulation methods, like tDCS, may offer insights into human performance when cognitive and perceptual enhancement are beneficial. This review synthesizes research on how targeting various brain regions via tDCS can influence outcomes in interactive driving and flight simulators.

Driving studies indicate that anodal tDCS over the DLPFC affects lateral and vertical lane positioning when following a lead vehicle (Beeli et al., 2008; Facchin et al., 2023; Sakai et al., 2014). These findings suggest that tDCS can acutely impact operational (automatic, reactive) and maneuvering (controlled, tactical) driving behaviors (Michon, 1985). It is also likely that tDCS can influence strategic (goal-directed, proactive) driving behaviors, such as trip planning, route memory, or adapting to detours (Michon, 1985). Expanding the complexity of tasks to include strategic and goal-directed elements could be one approach to enhance functional and psychological fidelity, thereby bolstering task realism and immersion (Roberts A. P. J. et al., 2020). Defining meaningful performance benchmarks in driving simulators can further aid in translating research findings into practical, everyday use (McManus et al., 2024).

Similarly, the flight studies demonstrate that anodal tDCS over the DLPFC and PPC is associated with smoother landings, supported by converging neurophysiological evidence from EEG, fNIRS, and fMRI (Choe et al., 2016; Feltman and Kelley, 2024; Mark et al., 2023). Although landing is one of the most challenging tasks for pilots, most of the time spent in flight involves monitoring system controls, including autopilot. For example, future studies may wish to explore the effects of tDCS during monotonous monitoring tasks. This inquiry becomes even more interesting when considering that visual scanning strategies are modulated by expertise (Lefrancois et al., 2016; Lounis et al., 2021). Combining stimulation with multimodal training may enhance its effects (Ward et al., 2017) and contribute further to research on the long-term impacts of tDCS.

Several factors should be carefully considered when interpreting these findings and designing future research. Stimulation protocols must be optimized to reduce individual variability and potential biases. Within-group designs, sham controls, double-masking procedures, and carefully worded materials are some ways to address participant and experimenter biases. It is also critical to address systematic racial bias in neurophysiological research. Most studies in this review recruited small samples of young men, and participants’ race or ethnicity was not reported. This omission raises concerns about inclusivity and generalizability, as methods that require adherence between electrodes and the scalp often exclude individuals based on hair type and style (Choy et al., 2022; Parker and Ricard, 2022; Roberts S. O. et al., 2020). Diverse, representative samples are essential to extend research beyond the lab and achieve broader inclusivity.

In summary, tDCS has the potential to modulate brain activity in regions that facilitate vehicle operation on the ground and in the clouds. To deepen our understanding of neuromodulation for human enhancement and continue exploring its possibilities, it is crucial to design stimulation protocols that mitigate biases and conduct studies with tasks or environments that reflect real-world conditions. As the promise of tDCS grows, it is essential to conduct rigorous investigations to fully understand its implications and optimize its application in various contexts.
